# Molecular Dynamics and Near-*T_g_* Phenomena of Cyclic Thioethers

**DOI:** 10.3390/ijms242417166

**Published:** 2023-12-06

**Authors:** Hubert Hellwig, Andrzej Nowok, Paulina Peksa, Mateusz Dulski, Robert Musioł, Sebastian Pawlus, Piotr Kuś

**Affiliations:** 1 Center for Integrated Technology and Organic Synthesis (CiTOS), MolSys Research Unit, University of Liège, B6a, Room 3/19, Allée du Six Août 13, 4000 Liege, Belgium; hhellwig@uliege.be; 2 Department of Experimental Physics, Wrocław University of Science and Technology, Wybrzeże Stanisława Wyspiańskiego 27, 50-370 Wrocław, Poland; andrzej.nowok@pwr.edu.pl (A.N.); paulina.peksa@pwr.edu.pl (P.P.); 3 Faculty of Science and Technology, Institute of Materials Engineering, University of Silesia in Katowice, 75 Pułku Piechoty 1A, 41-500 Chorzów, Poland; mateusz.dulski@smcebi.edu.pl; 4 Institute of Chemistry, University of Silesia in Katowice, Szkolna 9, 40-003 Katowice, Poland; pkus@ich.us.edu.pl; 5 August Chełkowski Institute of Physics, University of Silesia in Katowice, 75 Pułku Piechoty 1, 41-500 Chorzów, Poland; sebastian.pawlus@us.edu.pl

**Keywords:** glass transition, supercooled liquid, crown ethers, thioethers, dielectric spectroscopy, relaxation process, vitrification

## Abstract

This article presents the synthesis and molecular dynamics investigation of three novel cyclic thioethers: 2,3-(4′-methylbenzo)-1,4,7,10-tetrathiacyclododeca-2-ene (compound **1**), 2,3,14,15-bis(4′,4″(5″)-methylbenzo)-1,4,7,10,13,16,19,22,25-octathiacyclotetracosa-2,14-diene (compound **2**), and 2,3,8,9-bis(4′,4″(5″)-methylbenzo)-1,4,7,10-tetrathiacyclododeca-2,8-diene (compound **3**). The compounds exhibit relatively high glass transition temperatures (*T_g_*), which range between 254 and 283 K. This characteristic positions them within the so-far limited category of crown-like glass-formers. We demonstrate that cyclic thioethers may span both the realms of ordinary and sizeable molecular glass-formers, each featuring distinct physical properties. Furthermore, we show that the *T_g_* follows a sublinear power law as a function of the molar mass within this class of compounds. We also reveal multiple dielectric relaxation processes of the novel cyclic thioethers. Above the *T_g_*, their dielectric loss spectra are dominated by a structural relaxation, which originates from the cooperative reorientation of entire molecules and exhibits an excess wing on its high-frequency slope. This feature has been attributed to the Johari–Goldstein (JG) process. Each investigated compound exhibits also at least one intramolecular secondary non-JG relaxation stemming from conformational changes. Their activation energies range from approximately 19 kJ/mol to roughly 40 kJ/mol. Finally, we analyze the high-pressure molecular dynamics of compound **1**, revealing a pressure-induced increase in its *T_g_* with a *dT_g_*/*dp* coefficient equal to 197 ± 8 K/GPa.

## 1. Introduction

Crown and heterocrown ethers constitute one of the most vital classes of heterocyclic molecules in chemistry, science and industry. Since their first pioneering synthesis by Pedersen, which earned him a Nobel Prize [1], crown ethers have experienced a research explosion leading to an in-depth understanding of their versatile conformational interconversion possibilities, selective complexation of ions, and structure–property relationships among their host–guest hybrid compounds [2,3,4,5,6]. Even sought-after properties, such as ferroelectricity, ferroelasticity, piezoelectricity, temperature-induced dielectric and optical switching, have been identified in the latter case [7,8,9,10,11,12,13]. Consequently, crown-like compounds have rapidly gained recognition as heterocycles with immense and versatile application possibilities, e.g., in ionic sensing, treatment of nuclear waste, phase transfer catalysis, pharmacy engineering, drug delivery, biotechnology (as artificial ion channels), electronics, and others [14,15,16,17,18,19,20,21,22]. Molecular dynamics studies play a pivotal role in these fields, offering direct insight into the mechanisms and time scales of the physical processes occurring in the systems [23,24,25,26]. They also enable the analysis of amorphous phase stability and tendencies toward recrystallization, which are crucial aspects of the potential applications of crown ethers and cyclic thioethers in drug delivery and design [22,26]. Nevertheless, many issues remain unresolved concerning the molecular dynamics and vitrification of crown ethers and heterocrowns.

For instance, for many years, compounds with this chemical structure have been predominantly regarded as crystalline substances [27,28,29,30,31,32]. Indeed, most of them crystallize with ease, and instances of their glass-forming representatives remain few and far between [33,34]. Notably, the driving forces that underlie such high crystallization propensity are still elusive, especially considering the remarkable flexibility of the heterocyclic crown-like ring. For example, 18-crown-6 may adopt over a thousand distinct conformations, further complicating the matter [35,36]. In both the liquid and supercooled liquid states, crown-like molecules tend to self-organize into small intermolecular structures [34,37]. This process becomes more pronounced as the temperature decreases [34,37]. Nevertheless, the exact architecture of the molecular clusters and their potential relationship with the crystallization or vitrification tendencies remain unknown for crown ethers and their analogues. Finally, there are still notable gaps and inconsistencies in our understanding of the molecular dynamics and near-*T_g_* phenomena among crown-like glass-formers. While some extraordinary characteristics have been proposed for 15-crown-5 (including the presence of multiple calorimetric endotherms directly linked to its rich dielectric response), other research indicates behavior in heterocrowns that aligns more closely with the typical traits of van der Waals glass-formers [33,34].

To bridge the existing gap, we synthesize three novel glass-forming cyclic thioethers and thoroughly investigate their molecular dynamics. These compounds are: 2,3-(4′-methylbenzo)-1,4,7,10-tetrathiacyclododeca-2-ene (compound **1**), 2,3,14,15-bis(4′,4″(5″)-methylbenzo)-1,4,7,10,13,16,19,22,25-octathiacyclotetracosa-2,14-diene (compound **2**), and 2,3,8,9-bis(4′,4″(5″)-methylbenzo)-1,4,7,10-tetrathiacyclododeca-2,8-diene (compound **3**). They are analogues of the previously reported 6-methyl-2,3-dihydro-1,4-benzodithiine and 2,3-(4′-methylbenzo)-1,4-dithia-7-oxacyclononane), which will be further abbreviated as **MeBzS_2_** and **MeBzS_2_O**, respectively [34]. Thus, our study extends the understanding of the physics and molecular dynamics of cyclic thioethers. Using differential scanning calorimetry (DSC) and broadband dielectric spectroscopy (BDS), we unveil the relatively high *T_g_* of compounds **1**–**3**, which range between 254 and 283 K. In this respect, we show that the *T_g_* generally follows a sublinear power law as a function of the molar mass within the entire class of cyclic thioethers. Additionally, we delve into the rich dielectric response of compounds **1**–**3**, identifying multiple relaxation phenomena, elucidating their origin, and determining the temperature dependences of related relaxation times. We report three well-resolved relaxation processes for compound **1**: the structural α relaxation and two intramolecular secondary processes, β and γ. In turn, we observe two well-resolved relaxations (α and β) for compounds **2** and **3**. By employing the Coupling Model and high-pressure dielectric measurements, we unveil the intramolecular character of the β processes in all the studied compounds and discuss the surprising discrepancy in their nature from the β relaxation of the previously reported **MeBzS_2_O**. We also answer the question of whether these compounds exhibit some extraordinary feature or behave rather like typical van der Waals liquids.

## 2. Results

**Figure 1:**
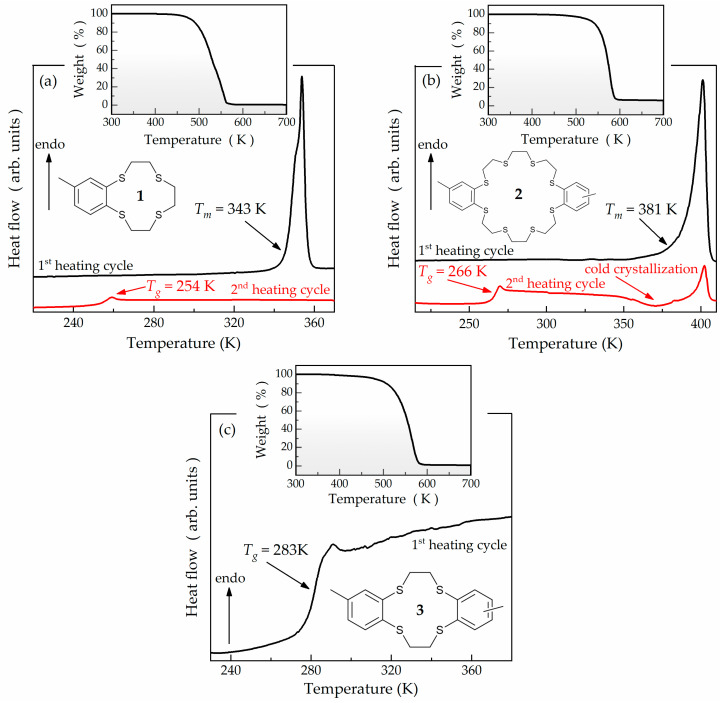
Chemical structures of the studied compounds **1** (**a**), **2** (**b**), and **3** (**c**), along with their DSC thermograms collected while heating at a rate of 10 K/min. Upper insets show weight losses observed during the TGA measurements.

Compounds **1**–**3** are cyclic thioethers, each featuring at least one aromatic ring (Figure 1a–c). A common structural element among them is the presence of ethylene bridges (-CH_2_-CH_2_-) that connect the sulfur atoms in the heterocyclic ring. Specifically, this ring comprises 12 atoms with 4 sulfur atoms in the case of compounds **1** and **3**, and 24 atoms with 8 sulfur atoms in the case of compound **2**. Notably, compound **2** stands out as an analogue of compound **1**, boasting a heterocyclic ring twice the size and a doubled number of aromatic moieties and molar mass compared to compound **1**. Compound **3** is a similar analogue to the previously reported **MeBzS_2_** [34]. Compounds **1**–**3** are commercially unavailable and have been synthesized by us following the procedure described in the Materials section and Supplementary Information (SI).

Our characterization of compounds **1**–**3** starts with thermogravimetry (TGA) and DSC studies, following the protocols described in the Experimental section. As revealed using the TGA technique, each compound is stable up to 450 K (see insets of Figure 1a–c). Initial weight losses of 5% occur at approximately 480, 524, and 483 K for compounds **1**, **2**, and **3**, respectively. Consequently, any thermal effect observed via calorimetry below these conditions should be attributed to a phase transition. The resulting thermograms from the heating cycles are depicted in Figure 1a–c. It is evident that compounds **1** and **2** initially take the form of crystalline solids (see the black curves in Figure 1a,b). Their melting points are approximately 343 and 381 K and manifest as distinct endothermal peaks on the thermograms. Compounds **1** and **2** do not recrystallize upon cooling from the melted state. Instead, they readily undergo vitrification. This remarkable glass-forming ability is observed in their second thermograms acquired while heating at a rate of 10 K/min (see the red curves in Figure 1a,b). These thermograms exhibit a thermal effect related to the glass transition, which occurs at approximately 254 K for compound **1** and 266 K for compound **2**. Surprisingly, despite its larger molecular size and higher number of single bonds within the heterocyclic ring, compound **2** exhibits a higher tendency to recrystallize than compound **1**. This increased crystallization tendency is evident in the exothermal effect seen in the second thermogram of compound **2** at around 363 K (see the red curve in Figure 1b). Owing to the substantial heating rate (10 K/min), crown-like macrocycle **2** only partially recrystallized from the supercooled liquid state during the experiment, which is reflected in a less pronounced melting peak. Contrary to compounds **1** and **2**, crown-like heterocycle **3** forms a viscous liquid at 298 K after synthesis (Figure 1c). It undergoes vitrification at roughly 283 K, which is discernible as a thermal effect on the corresponding thermogram. To shed more light on the glass-forming properties of the cyclic thioethers in question, we investigate them using the BDS technique.

**Figure 2:**
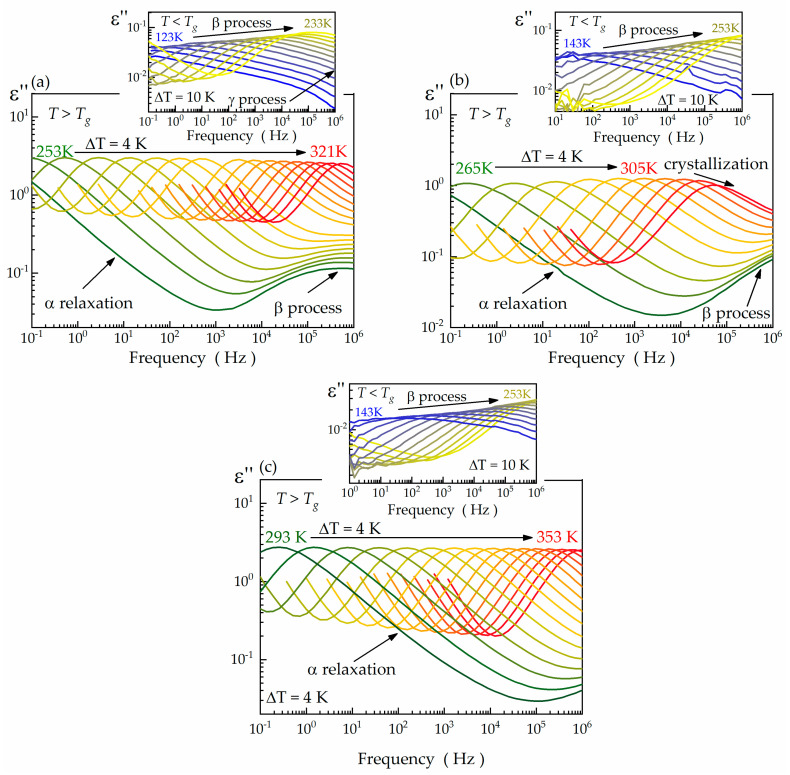
Dielectric loss spectra of compounds **1** (**a**), **2** (**b**), and **3** (**c**).

Figure 2a–c depict the selected frequency-dependent dielectric loss spectra, *ε*″(*f*), of compounds **1**–**3** collected above and below their respective *T_g_* values. Similar to their previously reported analogues [34], the dielectric response of the cyclic thioethers **1**–**3** also comprises structural and secondary relaxation processes. The maxima of the structural α-process remain visible exclusively above *T_g_* within the frequency window of 10^−1^–10^6^ Hz. This is because the α relaxation is related to the cooperative relaxation dynamics of entire molecules in the liquid phase [38], and the glass transition occurs when the structural relaxation time (*τ_α_*) reaches 100 s, which corresponds to a frequency of 1.59 × 10^−3^ Hz. The structural α-relaxation of compounds **1**–**3** manifests as asymmetric bell-shaped loss peaks in the *ε*″(*f*) spectra that a single Havriliak–Negami fit function can effectively parametrize:

(1) $$\varepsilon^{*}=\varepsilon_{\infty }+\frac{\Delta \varepsilon }{{\left(1+{\left(i\omega \tau_{HN}\right)}^{\alpha_{HN}}\right)}^{\beta_{HN}}},$$

in which ε* is the complex dielectric permittivity, *ε_∞_*—the high-frequency limit of dielectric permittivity, Δ*ε*—dielectric strength, *ω*—angular frequency, *τ_HN_*—the so-called Havriliak–Negami relaxation time, and *α_HN_*, *β_HN_* are shape parameters describing the symmetric and asymmetric broadening of the relaxation process [39]. The typical *α_HN_*, *β_HN_* values of the α relaxation fall within the following ranges: *α_HN_* ∈ (0.88; 0.96) and *β_HN_* ∈ (0.57; 0.63) for compound **1**, *α_HN_* ∈ (0.82; 0.90) and *β_HN_* ∈ (0.52; 0.60) for compound **2**, and *α_HN_* ∈ (0.88; 0.92) and *β_HN_* ∈ (0.54; 0.62) for compound **3**.

The secondary relaxations in compounds **1**–**3** are predominantly observed below the *T_g_* within the studied frequency range (Figure 2a–c). Compounds **1** and **2** exhibit a single well-resolved secondary process, called β relaxation. In turn, compound **3** is characterized by two distinct secondary relaxations (β and γ) with different amplitudes and peak positions. Their common denominator is the broad and symmetric shape of the relaxation loss peaks that can be well described utilizing Cole–Cole formalism [40]:

(2) $$\varepsilon^{*}=\varepsilon_{\infty }+\frac{\Delta \varepsilon }{1+{\left(i\omega \tau_{CC}\right)}^{\alpha_{HN}}}.$$

In this formula, *τ_CC_*—the so-called Cole–Cole relaxation time, and the meaning of other parameters remains unchanged with respect to the Havriliak–Negami function [40]. Notably, the difference between both expressions lies in the shape parameter *β_HN_*, which takes the fixed value of 1 in the case of Cole–Cole formalism. The secondary relaxations become narrower as the temperature increases, which is reflected in their shape parameter *α_HN_*. In the case of the β process, the *α_HN_* increases from ~0.2 to ~0.5 for compounds **1** and **2**, and from ~0.18 to ~0.35 for compound **3**. The *α_HN_* value of the γ relaxation of compound **1** ranges between 0.21 and 0.24.

The fitting parameters obtained for the α, β, and γ processes have been further used to calculate their corresponding relaxation times *τ_α_*, *τ_β_*, *τ_γ_* based on the following formulas [38]:

(3) $$\tau_{\alpha }=\tau_{HN}[sin(\frac{\alpha_{HN}\pi }{2\beta_{HN}+2}){]}^{-\frac{1}{\alpha_{HN}}}[sin(\frac{\alpha_{HN}\beta_{HN}\pi }{2\beta_{HN}+2}){]}^{\frac{1}{\alpha_{HN}}};$$

(4) $$\tau_{\beta ,\gamma }=\tau_{CC}$$

**Figure 3:**
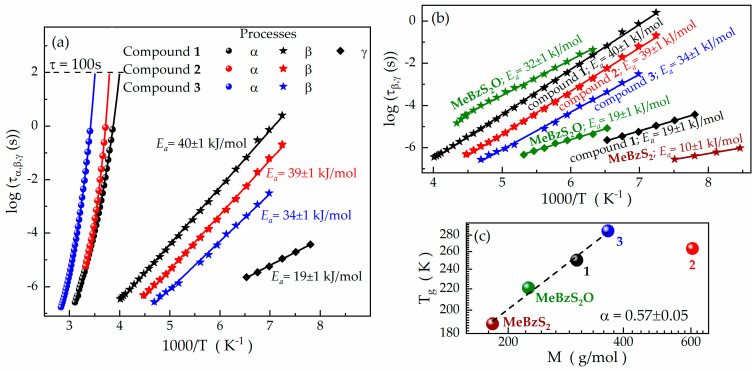
(**a**) Temperature dependences of the relaxation times *τ_α_*, *τ_β_*, *τ_γ_* of compounds **1**–**3**. (**b**) Comparison between the times of the secondary relaxations in compounds **1**–**3** and their previously reported analogues **MeBzS_2_O** and **MeBzS_2_**. The β and γ relaxations are marked by stars and diamonds, respectively. The *τ_β_* and *τ_γ_* are reproduced from Ref. [34] with permission from the Royal Society of Chemistry. (**c**) The relationship between the *T_g_* and molar mass among the cyclic thioethers.

Their ambient-pressure temperature dependences are presented in Figure 3a. According to this plot, each log*τ_α_* = *f*(1000/*T*) relationship conforms to a non-linear, super-Arrhenius pattern, which can be effectively parametrized using the Vogel–Fulcher–Tammann (VFT) formula:

(5) $$\tau_{\alpha }\left(T\right)=Aexp\left(\frac{B}{T-T_{0}}\right),$$

where *A*, *B*, and *T*_0_ denote a pre-exponential factor, a material constant, and the ideal glass temperature, respectively [41,42,43]. This dependence is the steepest near the glass transition for each cyclic thioether. To quantify the slowing of the relaxation times near the *T_g_*, we calculate the values of the so-called *fragility* index *m_p_* according to the formula [38]:

(6) $$m_{p}={\left.\frac{dlog_{10}\tau_{\alpha }}{d\left(T_{g}/T\right)}\right|}_{T=T_{g}},$$

where *T_g_* is considered a temperature at which *τ_α_* equals 100 s. As summarized in Table 1, *m_p_* assumes values greater than 30 for all the cyclic thioethers and even exceeds 100 in the case of compound **1**. Following Angell’s concept [44], the obtained values allow us to categorize the compounds in question as moderately fragile (compounds **1** and **3**) or fragile liquids (compound **2**). The *T_g_*s determined from dielectric studies according to this definition are close to the values obtained from the DSC measurements (see Table 1). The slight discrepancy may result from different experimental protocols: while calorimetric thermograms were collected with a heating rate of 10 K/min, the dielectric measurements were conducted under quasi-static conditions (i.e., after prior temperature stabilization).

In contrast to the *τ_α_*, the relaxation times *τ_β_* and *τ_γ_* (in their logarithm form) exhibit linear variation with 1000/T, adhering to the Arrhenius law:

(7) $$\tau_{\beta }=\tau_{0}exp\left(\frac{E_{a}}{RT}\right).$$

Here, *R*, *E_a_*, and *τ*_0_ represent the gas constant, the activation energy, and the relaxation time at the limit of an infinitely high temperature, respectively [38]. The parameter values obtained for the secondary relaxations of compounds **1**–**3** are summarized in Table 2.

As presented above, the *E_a_* values (19–40 kJ/mol) fall within the typical range for secondary processes in van der Waals liquids. Additionally, their *E_a_*/(*RT_g_*) ratio consistently remains significantly below 24. Consequently, in accordance with the concept of Kudlik et al. [45,46,47,48], we can categorize the β and γ relaxations of compounds **1**–**3** as intramolecular secondary processes. One can speculate that these processes are likely associated with field-induced conformational changes within the heterocyclic ring, as previously reported for two analogous compounds [34]. Indeed, the *E_a_* values of the γ relaxations are precisely the same for compound **1** and the previously reported **MeBzS_2_O** (see Figure 3b). Their relaxation times are also comparable, which is particularly well illustrated at ~153 K, where *τ_γ_* takes the value of 2.3 μs for compound **1** and 8.3 μs for **MeBzS_2_O** (Figure 3b). Considering the similar chemical structures of both compounds, with one aromatic ring and -CH_2_-CH_2_- bridges linking the heteroatoms, these features strongly indicate that the physical origin of the γ relaxation is similar (or even the same) in both molecules.

The β relaxation times also exhibit resemblances between compound **1** and the previously reported **MeBzS_2_O** (Figure 3b). Specifically, at approximately 158 K, *τ_β_* is equal to 23 ms for compound **1** and 43 ms for **MeBzS_2_O**. However, the β relaxation of the latter cyclic thioether has been identified as a Johari–Goldstein (JG) secondary process and associated with the limited reorientation of entire molecules [34]. To elucidate the physical origin of the β processes in the studied compounds **1**–**3**, we employ the Coupling Model. According to this model, the relaxation time of the secondary JG process, *τ_JG_*, is correlated with *τ_α_* by the following formula:

(8) $$\tau_{JG}\left(T\right)≅\tau_{0}\left(T\right)=t_{c}^{n}\tau_{\alpha }{\left(T\right)}^{1-n}.$$

In this equation, *τ*_0_ is the ′primitive′ relaxation time, *n* is the coupling parameter, and *t_c_* is the crossover time from independent relaxation to cooperative relaxation equal to 1–2 ps [49,50]. The parameter *n* is, in turn, linked to the *β_KWW_* factor of the Kohlrausch–Williams–Watts (KWW) function *ϕ*(*t*) [51] by the relationship $n=1-\beta_{KWW}$:

(9) $$\phi \left(t\right)=exp\left[-{\left(\frac{t}{\tau_{\alpha }}\right)}^{1-n}\right].$$

Therefore, as described earlier [52], we use a one-side Fourier transform of the KWW function to fit the α relaxation in the low-temperature dielectric spectra of compounds **1**–**3**:

(10) $$\varepsilon^{*}\left(\omega \right)=\Delta \varepsilon \int_{0}^{\infty }e^{i\omega t}\left[-\frac{d}{dt}exp\left(-{\left(\frac{t}{\tau_{\alpha }}\right)}^{1-n}\right)\right]dt.$$

**Figure 4:**
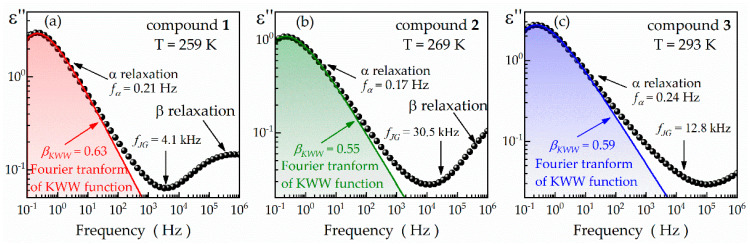
One-side Fourier transform KWW functions superimposed on the exemplary dielectric loss spectra, collected at 259 K for compound **1** (**a**), 269 K for compound **2** (**b**), and 293 K for compound **3** (**c**).

Specifically, we apply this methodology to the spectra collected at 259, 269 and 293 K for compounds **1**, **2**, and **3**, respectively, when the α relaxation peak was at approximately 0.2 Hz (see Figure 4a–c). Under these conditions, the relaxation times *τ_α_* are 0.74 s for compound **1**, 0.93 s for compound **2**, and 0.65 s for compound **3**. As shown in Figure 4a–c, the KWW function fits well with the α relaxation peak, mainly near the maximum, which is typical of molecular glass-formers. The best fits are obtained for the *β_KWW_* parameter equal to 0.63, 0.55, and 0.59 for compounds **1**, **2**, and **3**, respectively. Applying Formula (8) to the *τ_α_* and *β_KWW_* values, we determine the *τ*_0_ parameter as 38.9 μs, 5.2 μs and 12.4 μs for compounds **1**, **2**, and **3**, respectively. Consequently, one can expect to detect the JG secondary relaxation at *f*_0_ equal to roughly 4.1 kHz for compound **1**, 30.5 kHz for compound **2**, and 12.8 kHz for compound **3**. For all the cyclic thioethers, these values do not coincide with the location of the β relaxation maximum, affirming their prior classification as intramolecular non-JG relaxations [53]. The obtained *f*_0_ value for compound **3** correlates well with the excess wing of its α relaxation (see Figure 4c). Therefore, it is plausible that the secondary JG relaxation in all the compounds **1**–**3** takes is located in the area of the excess wing at ambient pressure. Unfortunately, this defining characteristic is not discernible for compounds **1** and **2** due to the significant contributions of their non-JG β relaxations. Consequently, we perform high-pressure dielectric studies on the exemplary compound **1** to validate our hypothesis.

**Figure 5:**
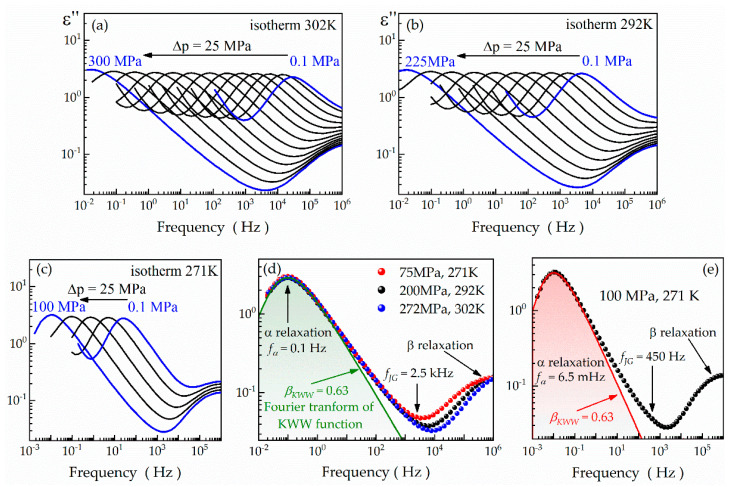
Representative dielectric loss spectra collected during isothermal compression of compound **1** at 302 K (**a**), 292 K (**b**), and 271 K (**c**). (**d**) High-pressure *ε″*(*f*) spectra measured under isochronous conditions for compound **1** while maintaining the α relaxation peak at 0.1 Hz. (**e**) One-side Fourier transform KWW functions superimposed on the exemplary dielectric loss spectrum collected at 271 K and 100 MPa for compound **1**, revealing the non-JG character of its β relaxation.

Figure 5a–c illustrate the dielectric loss spectra registered during the isothermal compression of compound **1** at 302, 292, and 271 K. The common trend in these spectra is the gradual shift of the α relaxation toward lower frequencies as the pressure increases. This phenomenon results from the stepwise retardation of molecular dynamics due to pressure-induced increases in viscosity, density, and, as a result, a reduction in the free volume between molecules [54]. This occurs despite the thermal energy delivered to the system remaining unchanged under isothermal conditions. In contrast to the α process, the secondary β relaxation of compound **1** appears unaffected by the applied pressure (Figure 5a–c). This is particularly evident at 271 K, when the β relaxation peak is positioned at approximately 1 MHz (Figure 5c). This feature is another distinctive characteristic of intramolecular non-JG secondary relaxations in molecular glass-formers [54]. To gain further insights, we perform high-pressure dielectric measurements under isochronous conditions, maintaining the α relaxation peak at around 0.1 Hz. Such conditions can be achieved only at specific pressure and temperature values, including 75 MPa and 271 K, 220 MPa and 292 K, 272 MPa and 302 K. As presented in Figure 5d, the β relaxation of compound **1** changes its position under these conditions, despite the preserved shape of the α relaxation peak and its consistent location at roughly 0.1 Hz. This finding provides additional evidence of the non-JG character of the β relaxation in compound **1** because of the disagreement of the observed trend with the generalized Coupling Model [53]:

(11) $$\tau_{JG}\left(T,p\right)≅\tau_{0}\left(T,p\right)=t_{c}^{n}\tau_{\alpha }{\left(T,p\right)}^{1-n}.$$

Namely, under these conditions, the α relaxation peak can be fitted to the KWW function with *β_KWW_* = 0.63, consistent with the value observed at ambient pressure. Applying Equation (11), we obtain a constant value of the *τ*_0_ parameter (*τ*_0_ = 38.9 μs) for all the pressure–temperature combinations mentioned before, which corresponds to *f*_0_ = 2.5 kHz. This value aligns well with the excess wing of the α relaxation (Figure 5d). This correlation is even better visible in the representative dielectric spectrum registered under 100 MPa at 271 K, where the α relaxation peak is at roughly 6.5 mHz (Figure 5e). Here, the *f*_0_ parameter takes the value of approximately 450 Hz, corresponding to the excess wing of the α relaxation. Therefore, we can conclude that the secondary JG relaxation is manifested as an excess wing also for compound **1**. However, unlike compound **3**, it remains discernible only under higher pressure conditions due to the more significant contributions of the non-JG β relaxation.

To complete the discussion of the high-pressure molecular dynamics of compound **1**, we fit the α relaxation process in all the collected spectra utilizing Equation (1) with an added dc conductivity term:

(12) $$\varepsilon^{*}=\frac{\sigma }{\varepsilon_{0}\omega }+\varepsilon_{\infty }+\frac{\Delta \varepsilon }{{\left(1+{\left(i\omega \tau_{HN}\right)}^{\alpha_{HN}}\right)}^{\beta_{HN}}}.$$

**Figure 6:**
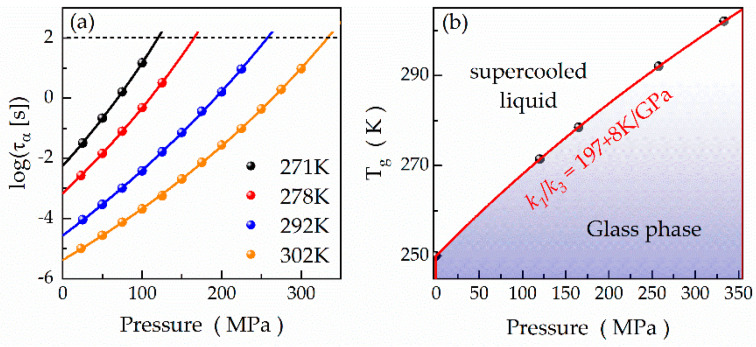
(**a**) Pressure dependences of *τ_α_* at selected temperatures for compound **1**. (**b**) Pressure-induced changes in the *T_g_* of compound **1**.

In this formula, *ε*_0_ is the vacuum permittivity, *ω* denotes the angular frequency, and the whole term $\frac{\sigma }{\varepsilon_{0}\omega }$ characterizes the conductivity contribution σ to the dielectric losses. As a result, we obtain similar values for the shape parameters *α_HN_* and *β_HN_* compared to the ambient-pressure conditions. Figure 6a illustrates the pressure dependences of the relaxation times *τ_α_*. According to this graph, *τ_α_* increases with rising pressure in a super-Arrhenius manner, typical of molecular glass-formers. These pressure dependences are well described by the pressure counterpart of VFT equation:

(13) $$\tau_{\alpha }\left(p\right)=\tau_{\alpha ,0.1\mathrm{MPa}}\cdot exp\left(\frac{D_{p}p}{p_{0}-p}\right),$$

where *τ_α,_*_0.1MPa_ denotes the ambient-pressure α relaxation time, *D_p_* is the so-called isothermal strength parameter, and *P*_0_ is the pressure of the ideal glass transition [54]. The parameters *τ_α,_*_0.1MPa_, *D_p_*, *P*_0_ for all the curves are summarized in Table 3. Extrapolating the pressure dependences *τ_α_*(*p*) to 100s allows us to construct the phase diagram for compound **1**. As illustrated in Figure 6b, the *T_g_* increases with pressure non-linearly. This dependence was parametrized with the phenomenological Anderson–Anderson equation:

(14) Tg=k11+k2k3P1k2,

where *k*_1_, *k*_2_, *k*_3_ are material constants, and the ratio of *k*_1_/*k*_3_ defines the pressure coefficient of the glass transition temperature at the limit of low pressures, d*T_g_*/d*p* (*k*_1_/*k*_3_ = d*T_g_*/d*p*) [54,55]. We determined the d*T_g_*/d*p* as 197 ± 8 K/GPa for compound **1**, which means that the elevation of the pressure by merely 100 MPa increases the *T_g_* by as much as ~20 K.

Recognizing the intramolecular nature of both the β and γ relaxations in compounds **1**–**3**, it becomes imperative to elucidate their physical origins. In analogous cases, such as **MeBzS_2_** and **MeBzS_2_O**, non-JG secondary relaxations have been attributed to interconversion between conformers [34]. This phenomenon was also manifested in the FTIR spectra as temperature-induced variations in the abundance of specific geometries [34]. Consequently, we perform similar temperature-dependent measurements for the representative compounds **1** and **2**.

**Figure 7:**
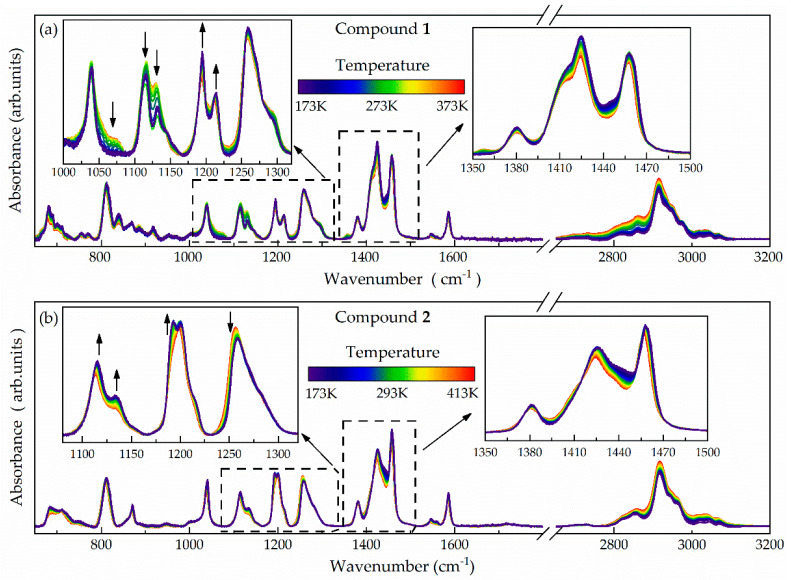
Infrared spectra of compounds **1** (**a**) and **2** (**b**) collected at various temperatures.

The FTIR spectra of compounds **1** and **2** have been collected in the 650–3600 cm^−1^ spectral range and contain numerous bands (Figure 7a,b). The range of 2700–3100 cm^−1^ encompasses stretching vibrations of CH_x_ groups (where x = 1, 2, 3), including the benzene *ν*CH, aliphatic ethylene *ν*CH_2_, and methyl *ν*CH_3_. However, our primary focus in this analysis is IR-active vibrations spread over the fingerprint region (i.e., 650–1700 cm^−1^), which contains (among others) characteristic modes of the benzene ring, aliphatic ethylene groups -CH_2_-CH_2_, *δ*CH_2_ within the aliphatic sulfide groups -CH_2_-S-, the deformational *δ*CH_3_ within Ar-CH_3_ (where Ar denotes an aromatic ring) or vibrations of the -S-Ar moieties, further confirming the chemical structure of the investigated compounds [56]. In this spectral range, the temperature-dependent experiments have revealed slightly distinct spectral behavior for individual bands for both compounds **1** and **2**.

As presented in Figure 7a, the FTIR spectra of compound **1** display a reversible trend of temperature-induced changes, with a slight modification in the line shape and reduction in the intensity of the bands at ~1000–1160 cm^−1^ concurrent with an increase in the intensity of the bands at ~1160–1230 cm^−1^ as the temperature decreased. These alterations correspond to a slight shift toward higher wavenumbers and an increase in the intensity of the bands around 1370–1500 cm^−1^. A characteristic feature of compound **1** is also the gradual vanishing of the bands at roughly 1070 cm^−1^ and 1360 cm^−1^ while cooling. In contrast, the temperature-dependent FTIR spectra of compound **2** exhibit a lower, albeit noticeable, increase in the intensity of all the bands spreading over 1000–1230 cm^−1^, with a slight modification of their line shape (Figure 7b). The changes are accompanied by a decrease in the intensity of the band at ~1260 cm^−1^ and a slight shift of the bands around 1370–1500 cm^−1^. According to the literature, the most intense temperature-induced changes occur mainly for bands related to the moieties of the heterocyclic thioether ring in both compounds [56]. These interrelated changes cannot be explained by simple density changes within the liquid phase, pointing to a gradual shift in the thermal equilibrium between several conformers. Consequently, they indicate the ongoing conformational alterations within the heterocyclic ring of the studied compounds, reinforcing the hypothesis of the intramolecular, conformational-related origin of the dielectric secondary relaxations observed for them.

## 3. Discussion

Compounds **1**–**3** belong to the group of cyclic thioethers and show a significant propensity for vitrification. Including these compounds, five macrocycles synthesized based on the 3,4-toluenedithiol building block have been identified as glass-formers [34]. A common structural feature among all glass-forming cyclic thioethers is a flexible heterocyclic ring. Notably, the herein-studied compound **2** possesses the highest number of single bonds within its heterocyclic ring, implying greater conformational diversity. However, paradoxically, it displays the highest tendency toward cold crystallization among compounds **1**–**3**. A significant crystallization tendency has also been observed in the case of **MeBzS_2_**, a crown-like analogue with a smaller, much stiffer, six-membered heterocyclic ring. Conversely, the cold crystallization process has not been observed at ambient pressure for compounds **1** and **3** (studied herein), nor for **MeBzS_2_O** [34]. Consequently, there may be no straightforward relationship between the cold crystallization propensity and the heterocyclic ring size among crown-like compounds. This statement is also supported by the fact that the energy barrier for the conformational interconversion of all the mentioned crown-like glass-formers falls within the range of 10–40 kJ/mol, which is lower than the energy barrier required to create substantial obstacles during the formation of crystallization nuclei (10 kcal/mol) [57]. Therefore, the so-far reported conformational changes with the energy barrier of 10–40 kJ/mol should not be considered a decisive factor regulating the crystallization propensity among the known crown-like glass-formers. However, studies on a larger group of cyclic thioethers are required to confirm this hypothesis.

In the realm of non-polymeric compounds, the value of *T_g_* is influenced by several factors, including the chemical composition, molar mass, the type of functional groups, and the rigidity of the molecular structure [58,59,60,61,62,63,64]. Compounds **1**–**3**, **MeBzS_2_** and **MeBzS_2_O** (reported previously [34]) also fall within this category of glass-formers. They feature only ether (-O-) or thioether (-S-) moieties within their heterocyclic rings, which are known to exert a negligible impact on the *T_g_* values. To better understand the role of the molar mass (*M*) in regulating the *T_g_* among the cyclic thioethers, we analyze the *T_g_* = *f*(*M*) dependence. As illustrated in Figure 3c, the *T_g_* generally increases with a rising *M* for these compounds, aligning with the well-established principle:

(15) $$T_{g}\left(M\right)\propto M^{\alpha },$$

where *α* is a power coefficient [58]. Compound **2** is the only one deviating from this trend. However, this cyclic thiacrown ether can be classified as a sizeable glass-former with a molar mass equal to 605 g/mol. In essence, sizable molecules are non-polymeric compounds characterized by molar masses of approximately 600 g/mol, number of atoms exceeding 80, and pre-exponential factor in the VFT equation significantly surpassing the phonon-like time-scale (10^−14^ s). Consequently, they bridge the gap between polymers and typical low-molecular-weight glass-forming systems, simultaneously exhibiting molecular properties dissimilar to their ‘classical’ non-sizeable counterparts [65,66,67]. All these criteria are satisfied by compound **2**. It is also worth noting that numerous deviations from Equation (15) have been reported for higher-molecular-weight glass-formers. For example, the *T_g_* tends to saturate above a specific molecular weight for polymeric systems, following the Fox–Flory equation [58]. Therefore, the unexpected deviation of compound **2** from Equation (15) is fully justified, considering the size of its molecules. One can also conclude that the molar mass is one of the most pivotal factors influencing the glass transition temperature among cyclic thioethers.

The behavior and properties of compounds **1**–**3**, **MeBzS_2_** and **MeBzS_2_O** are typical of van der Waals glass formers. The glass transition is evident in all these systems as a single thermal effect on the thermograms [34]. Furthermore, their dielectric response is dominated by structural *α* relaxation and secondary processes [34]. No additional transitions related to multiple relaxation modes (suggested before for other crown-like analogues [33]) have been observed in these cyclic thioethers. In terms of the secondary relaxations, compounds **1**–**3**, **MeBzS_2_** and **MeBzS_2_O** are characterized by well-resolved non-JG processes. Their physical origin can be ascribed to intramolecular conformational transformations within the heterocyclic thioether ring, the only non-rigid moiety in their structures. Such a mechanism was also suggested previously based on quantum DFT calculations. Compound **1** exhibits two intramolecular secondary relaxations (β and γ) with activation energies equal to 40 ± 1 and 19 ± 1 kJ/mol, respectively. Its γ relaxation closely resembles the non-JG γ process in **MeBzS_2_O** regarding the relaxation times and the *E_a_* value (the latter is the same in both compounds). Considering the similar chemical structures of both compounds, with one aromatic ring and -CH_2_-CH_2_- bridges linking heteroatoms, these features strongly indicate that the physical origin of the γ relaxation is similar (or even the same) in both molecules. The *E_a_* of the intramolecular β process in compound **1** is approximately twice the activation energy of the γ relaxation. Similar *E_a_* values were found for intramolecular non-JG β processes in compounds **2** and **3**. Each of them is significantly slower than the γ relaxation in compound **1**, **MeBzS_2_O** and the secondary relaxation in **MeBzS_2_** (see Figure 3b). These features suggest that the intramolecular β relaxations in compounds **1**–**3** originate from more complex structural transformations within the heterocyclic thiacrown ring. However, further studies are required in this area. Finally, it is noteworthy that the secondary JG process assumes the form of an excess wing in compounds **1**–**3**, faintly discernible in their dielectric loss spectra due to the substantial contribution of the non-JG β relaxations. The JG process becomes more pronounced under elevated pressure, as exemplified by our high-pressure studies on compound **1**. For this compound, a stepwise increase in the *T_g_* with rising pressure is observed. The coefficient d*T_g_*/d*p* takes the 197 ± 8 K/GPa value, which is comparable to non-associated van der Waals liquids or weakly associated systems [68,69]. This observation aligns with its chemical structure comprising chemical moieties that form only weak intermolecular interactions. Considering the reported self-organization processes in cyclic thioethers and other crown-like compounds, it is reasonable to infer that the formed intermolecular structures are likely small.

## 4. Conclusions

Three novel cyclic thioethers **1**–**3** have been synthesized and characterized utilizing differential scanning calorimetry and broadband dielectric spectroscopy. These compounds do not vitrify when cooled from the liquid state at a rate of 30 K/min. Moreover, they exhibit a relatively high glass transition temperature, which takes the value of approximately 254 K (compound **1**), 266 K (compound **2**), and 283 K (compound **3**). These characteristics position them within the so-far limited category of non-polymeric crown-like glass-formers, for which the glass transition temperature follows a sublinear power law as a function of the molar mass. We have also shown that compounds **1**–**3** behave like typical fragile and polar van der Waals glass-formers, with a single thermal effect related to the glass transition and dielectric response dominated by relaxation processes occurring both above and below *T_g_*. Their *fragility* index takes the value of 69, 113, and 88 for compounds **1**, **2**, and **3**, respectively. When examined in their supercooled liquid state, the dielectric loss spectra of compounds **1**–**3** are dominated by a pronounced structural process, connected with the cooperative reorientation of entire molecules. Its relaxation times conform closely to the Vogel–Fulcher–Tammann equation near *T_g_*. In contrast, the secondary relaxations of these compounds have intramolecular origin and exhibit an Arrhenius-like temperature dependence of the corresponding relaxation times. Compound **1** possesses two well-resolved secondary relaxations (β and γ) of intramolecular, non-JG character. Their activation energies *E_a_* are equal to 40 ± 1 kJ/mol and 19 ± 1 kJ/mol, respectively. In turn, compounds **2** and **3** are characterized by a single well-resolved secondary β relaxation, the *E_a_* parameter of which is equal to 39 ± 1 kJ/mol and 34 ± 1 kJ/mol, respectively. The physical origin of all these intramolecular secondary processes is most likely conformational changes within the heterocyclic thioether ring. The defining characteristic of compounds **1**–**3** is also the secondary JG relaxation manifesting as an excess wing of the α process. This process becomes more pronounced under elevated pressure, as exemplified by high-pressure studies on compound **1**. For this compound, the *T_g_* increases gradually with rising pressure, with d*T_g_*/d*p* equal to 197 ± 8 K/GPa. Lastly, the performed studies may suggest no simple correlation between the crystallization propensity and heterocyclic ring size of the cyclic thioethers.

## 5. Materials and Methods

### 5.1. Materials

Compounds **1**–**3** investigated in this research are cyclic thioethers, which are commercially unavailable. All the chemicals needed for their synthesis were obtained from well-recognized suppliers (Merck (Poznań, Poland), TCI (Łódź, Poland), Fisher Scientific (Warszawa, Poland), Chempur (Piekary Śląskie, Poland)) and were used without further purification. All the reactions were performed under an argon atmosphere. Preparative chromatographic separations were performed using Silica gel 60 and monitored using thin-layer chromatography (TLC Silica gel 60 F_254_). For the removal of high-boiling-point solvents, a standard rotary evaporator with an attached two-stage rotary-vane oil vacuum pump was sufficient.

### 5.2. Syntheses

**Figure 8:**
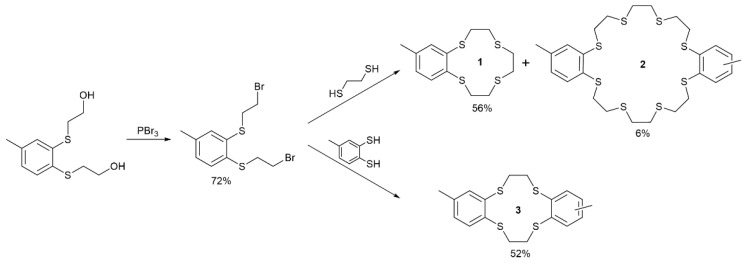
Synthesis scheme of compounds **1**, **2** and **3**.

Compounds **1**, **2**, and **3** were synthesized starting from 1,2-bis(2-bromoethyl)thio-4-methylbenzene, which is another compound not described before. This precursor was obtained via the reaction of 1,2-bis(2-hydroxyethylthio)-4-metylbenzene with PBr_3_ in DCM at 0 °C (Figure 8), and then purified by means of column chromatography, giving a yield of 72%. The 1,2-bis(2-hydroxyethylthio)-4-metylbenzene used for this synthesis was obtained from commercially available toluene-3,4-dithiol using a procedure described previously [70]. The cyclic thioethers **1** and **2** (i.e., (2,3-(4′-methylbenzo)-1,4,7,10-tetrathiacyclododeca-2-ene and 2,3,14,15-bis(4′,4″(5″)-methylbenzo)-1,4,7,10,13,16,19,22,25-octathiacyclotetracosa-2,14-diene) were synthesized simultaneously following the scheme presented in Figure 8. This reaction was conducted under high dilution conditions, using DMF as a solvent and cesium carbonate as a base. After chromatographic separation, compounds **1** and **2** were isolated with 56% and 6% yields. Compound **3** (2,3,8,9-bis(4′,4″(5″)-methylbenzo)-1,4,7,10-tetrathiacyclododeca-2,8-diene) was obtained under similar conditions, using toluene-3,4-dithiol in the place of 1,2-ethanedithiol. After chromatographic separation, compound **3** was isolated with a 52% yield. All the described here novel compounds were characterized via ^1^H and ^13^C NMR spectrometry. Detailed synthetic procedures and NMR spectra are included in the Supplementary Information.

### 5.3. NMR Spectroscopy

The ^1^H and ^13^C NMR spectra of compounds **1**–**3** and their precursors were recorded using a Bruker Avance 400 MHz spectrometer (Bruker, Rheinstetten, Germany), using CDCl_3_ (Cambridge Isotope Laboratories (Tewksbury, MA, USA)) as a solvent. The peaks were referenced to the residual CDCl_3_ resonances in the ^1^H and ^13^C NMR spectra, positioned at 7.28 and 77.04 ppm, respectively.

### 5.4. Thermogravimetry (TGA)

Thermogravimetric investigations of compounds **1**–**3** were conducted using a PerkinElmer Pyris 1 TGA instrument under a nitrogen gas atmosphere. The experimental conditions involved a temperature range 298 to 600 K, and a 15 K/min heating rate.

### 5.5. Differential Scanning Calorimetry (DSC)

Differential scanning calorimetry measurements of compounds **1**–**3** were performed using a Mettler-Toledo DSC 1 STARe System (Mettler Toledo, Columbus, OH, USA), which featured an intracooler and an HSS8 ceramic sensor equipped with 120 thermocouples. Before the calorimetric investigation, each sample was put into a sealed aluminum pan. The following experimental protocol was followed. In the first step, each sample was subjected to a heating scan with a rate of 10 K/min. The highest temperature was 373 K for compounds **1** and **3**, and 413 K for compound **2** (due its higher melting point). Subsequently, each melted sample was cooled down to 193 K at a rate of 30 K/min and kept at this temperature for 10 min. Finally, each compound was warmed up (to 373 K for compound **1** and **3**, or 413 K for compound **2**) with a rate of 10 K/min. All the investigations were conducted under a nitrogen atmosphere with a constant flow rate of 60 mL/min, and the thermograms were recorded only during heating scans.

### 5.6. Broadband Dielectric Spectroscopy (BDS) under Ambient Pressure

Broadband dielectric spectroscopy is a commonly employed technique for studying the molecular dynamics of simple glass-forming organic compounds. This technique was utilized by us also for compounds **1**–**3**. The ambient-pressure measurements covered wide frequency (10^−1^–10^6^ Hz) and temperature ranges. To perform the measurements, we used a stainless-steel parallel-plate capacitor equipped with two quartz spaces, each 100 μm thick, that created the necessary distance between the plates. Before the measurements, the capacitor was filled with a melted substance and sealed with a Teflon ring. The dielectric spectra were collected using a Novocontrol Broadband Dielectric Spectrometer (NOVOCONTROL Technologies GmbH & Co., KG, Montabaur, Germany) equipped with the Alpha Impedance analyzer. The desired temperature was stabilized with a precision better than 0.1 K using nitrogen gas and a Novocontrol Quattro system. Subsequent analysis of the recorded spectra was carried out using commercial WinFit software version 4.03 (NOVOCONTROL Technologies GmbH & Co., KG, Montabaur, Germany), exclusively in the representation of complex dielectric permittivity.

### 5.7. Broadband Dielectric Spectroscopy (BDS) under High Pressure

Dielectric experiments under elevated pressure were conducted using a high-pressure system provided by Unipress (Institute of High-Pressure Physics, Warszawa, Poland). Its crucial components were a high-pressure chamber (constructed from beryllium bronze) with a thermostatic mantle, a high-pressure closure with electric connections, a preliminary hand pump, and an automatic micropump (MP5 type) with a pressure controller. For these measurements, we utilized a stainless-steel parallel-plate capacitor with a diameter of 10 mm, the electrodes of which were separated by a Teflon spacer. Before the high-pressure investigations, the capacitor was filled with melted material (compound **1**), sealed and covered with Teflon tape to isolate it from the high-pressure medium (silicon oil of HL 80 type). The pressure was monitored using a Honeywell tensometric meter with a precision of 1 MPa, while the temperature was controlled by a Julabo Presto thermostatic bath (Seelbach, Germany) with a precision of 0.2 K. Isothermal measurements were conducted at 271, 278, 292 and 302 K. The dielectric spectra were recorded using a Novocontrol Broadband Dielectric Spectrometer equipped with an Alpha Impedance analyzer. Each spectrum was collected under quasi-static conditions after complete sample thermalization and at least 15 min of pressure stabilization.

### 5.8. Fourier Transform Infrared Spectroscopy (FTIR)

FTIR measurements were conducted using a Thermo Scientific IS50 spectrometer (Thermo Fisher Scientific, Madison, WI, USA) equipped with a standard source and a DTGS Peltier-cooled detector. For both compounds **1** and **2**, the process involved heating them above their respective melting points. The samples were placed between ZnSe glasses, with a 1 μm separation to ensure a consistent measuring chamber thickness. The spectra at each temperature were acquired by accumulating 32 scans with a spectral resolution of 4 cm^−1^, spanning the range of 650–4000 cm^−1^. Temperature-dependent measurements were carried out during the cooling process, covering the temperature range of 373 K (for compound **1**) or 413 K (for compound **2**) down to 173 K, with intervals of 10 K. Following the data collection, the experimental data underwent post-processing, which included baseline correction and the removal of interference from water and carbon dioxide.

## Figures and Tables

**Table 1:** Glass transition temperatures determined from calorimetric and dielectric studies, VFT fitting parameters and *fragility* index values for compounds **1**–**3**.

Compound	*T_g (DSC)_* (K)	*T_g (BDS)_* (K)	VFT Fitting Parameters			*m_p_*
			log(*A* (s))	*B* (K)	*T*_0_ (K)	
**1**	254	250	−13.2 ± 0.2	1940 ± 60	195 ± 2	69
**2**	266	263.5	−11.2 ± 0.2	940 ± 30	233 ± 1	113
**3**	283	285	−13.2 ± 0.1	1740 ± 40	236 ± 1	88

**Table 2:** Characteristic parameters describing the secondary relaxations of compounds **1**–**3**.

Compound	Relaxation	log(*τ*_0_ (s))	*E_a_* (kJ/mol)	*E_a_*/(*RT_g_*)
**1**	β	−15.0 ± 0.1	40 ± 1	19.2
	γ	−12.0 ± 0.1	19 ± 1	9.1
**2**	β	−15.6 ± 0.1	39 ± 1	17.8
**3**	β	−15.0 ± 0.1	34 ± 1	14.4

**Table 3:** Characteristic parameters describing the *τ_α_*(*p*) dependences of compound **1** at selected temperatures.

Temperature (K)	log(*τ_α,_*_0.1MPa_ (s))	*D_p_*	*P*_0_ (MPa)
271	−2.25 ± 0.02	46 ± 5	680 ± 60
278	−3.16 ± 0.01	46 ± 4	810 ± 60
292	−4.57 ± 0.03	53 ± 5	1100 ± 100
302	−5.38 ± 0.02	40 ± 2	1120 ± 40

## Data Availability

The data presented in this study are available on request from the corresponding author.

## Supplementary Materials

The following supporting information can be downloaded at https://www.mdpi.com/article/10.3390/ijms242417166/s1.
