# Supplementary material for: Molecular Dynamics and Near-Tg Phenomena of Cyclic Thioethers
Source: Int J Mol Sci. 2023 Dec 6;24(24):17166. doi: 10.3390/ijms242417166 (PMC10742681; doi:10.3390/ijms242417166)
Supplement: Supplementary file 1 [file ijms-24-17166-s001.zip › ijms-2717959-supplementary.pdf]

Molecular Dynamics and Near-T_g Phenomena of Cyclic Thioethers

Supplementary Information

Hubert Hellwig [1], Andrzej Nowok [2], Paulina Peksa [2], Mateusz Dulski [3], Robert Musioł [4,*], Sebastian Pawlus [5] and Piotr Kuś [6]

[1]*Center for Integrated Technology and Organic Synthesis (CiTOS), MolSys Research Unit, University of Liège, B6a, Room 3/19, Allée du Six Août 13, 4000 Liège, Sart Tilman, Belgium*

[2]*Department of Experimental Physics, Wrocław University of Science and Technology, Wybrzeże Stanisława Wyspiańskiego 27, 50-370 Wrocław, Poland*

[3]*Faculty of Science and Technology, Institute of Materials Engineering, University of Silesia in Katowice, 75 Pułku Piechoty 1A, 41-500 Chorzów, Poland*

[4]*Institute of Chemistry, University of Silesia in Katowice, 75 Pułku Piechoty 1A, 41-500 Chorzów, Poland*

[5]*August Chełkowski Institute of Physics, University of Silesia in Katowice, 75 Pułku Piechoty 1, 41-500 Chorzów, Poland*

[6]*Institute of Chemistry, University of Silesia in Katowice, Szkolna 9, 40-003 Katowice, Poland*

* robert.musiol@us.edu.pl

1. Experimental procedures for the synthesis of the investigated compounds

1.1. Synthesis of 1,2-bis(2-bromoethyl)thio-4-methylbenzene

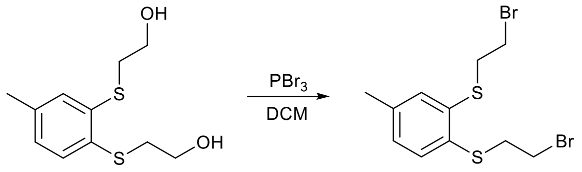

In a 100 ml round-bottom flask, 7.4 g (30.3 mmol) of 1,2-bis(2-hydroksyethylthio)-4-metylbenzene and 40 ml of DCM were placed. The flask was placed into an ice bath, and 2.0 ml (5.7 g, 21.0 mmol) of PBr_3 were added portion-wise within 10 minutes via syringe at 0 °C. After 6 h of stirring, 20 g of wate-ice were added to the flask. After melting of the ice, two-phase mixture was obtained. The phases were separated. Subsequently, the organic phase was washed with additional 20 ml of water and dried over $MgSO_4$. Crude product was purified using column chromatography (SiO_2, DCM:hexane, 4:1 by vol.), yielding 8.07 g (Y=72%) of slightly pink oil that solidifies after cooling to r.t.

M.p. = 52-54°C. ^{1}H NMR (400 MHz, $CDCl_3$) δ 7.29 (d, J = 7.8 Hz, 1H), 7.16 (d, J = 1.8 Hz, 1H), 7.04 (dd, J = 7.8, 1.8 Hz, 1H), 3.55 – 3.45 (m, 4H), 3.37 – 3.25 (m, 4H), 2.36 (s, 3H). ^{13}C NMR (101 MHz, $CDCl_3$) δ 138.34, 136.64, 131.74, 131.65, 130.52, 128.39, 35.83, 35.20, 29.79, 29.57, 21.15.

1.2. Synthesis of compounds 1 and 2 (2,3-(4'-methylbenzo)-1,4,7,10-tetrathiacyclododeca-2-ene; MeBz[12]aneS$_4$ and 2,3,14,15-bis(4',4"(5")-methylbenzo)-1,4,7,10,13,16,19,22,25-octathiacyclotetracosa-2,14-diene; bis(MeBz)[24]aneS$_8$)

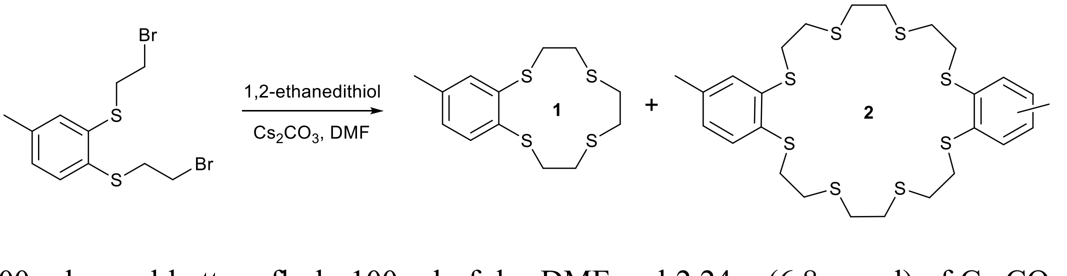

In a 500 ml round-bottom flask, 100 ml of dry DMF and 2.24 g (6.8 mmol) of Cs_2CO_3 were placed. The contents of the flask were kept at 55 °C. A solution of 1.48 g (4.0 mmol) 1,2-bis(2-bromoethylthio)-4-methylbenzene and 0.38 g (4.0 mmol) of 1,2-ethanedithiol in 70 ml of DMF were placed in an addition funnel and slowly added within 3 days. After that, the mixture was left stirring for a week. The mixture was filtered, solvent was removed using a rotary evaporator. Chromatographic separation (SiO_2, DCM:hexane, 1:1 vol.) yielded 0.72 g of compound **1** (Y=56%) and 0.07 g (Y=5.8%) of compound **2**.

Compound **1**: m.p.: 75-77 °C. ^{1}H NMR (400 MHz, $CDCl_3$) δ 7.28 (d, J = 8.1 Hz, 1H), 7.22 – 7.12 (m, 1H), 7.00 (dd, J = 8.0, 1.9 Hz, 1H), 3.35 (dt, J = 14.0, 6.6 Hz, 4H), 2.87 (s, 4H), 2.79 (dt, J = 17.8, 6.6 Hz, 4H), 2.35 (s, 3H). ^{13}C NMR (101 MHz, $CDCl_3$) δ 137.36, 136.41, 132.02, 131.55, 130.90, 127.79, 34.29, 34.20, 32.47, 32.16, 30.01, 29.86, 21.05.

Compound **2**: m.p.: 116-118 °C. ^{1}H NMR (400 MHz, $CDCl_3$) δ 7.28 (dd, J = 7.9, 3.9 Hz, 2H), 7.16 (dd, J = 4.5, 1.9 Hz, 2H), 7.01 (dt, J = 7.9, 1.0 Hz, 2H), 3.18 – 3.07 (m, 8H),z 2.89 – 2.67 (m, 16H), 2.35 (s, 6H). ^{13}C NMR (101 MHz, $CDCl_3$) δ 137.84, 137.74, 137.49, 137.29, 132.40, 132.19, 131.81, 131.55, 130.63, 130.36, 127.98, 127.89, 34.23, 34.13, 33.66, 33.54, 32.38, 32.32, 32.24, 32.20, 31.37, 31.31, 31.31, 31.24, 21.12, 21.12.

1.3. Synthesis of compound 3 (2,3,8,9-bis(4',4"(5")-methylbenzo)-1,4,7,10-tetrathiacyclododeca-2,8-diene; bis(MeBz)[12]aneS_4)

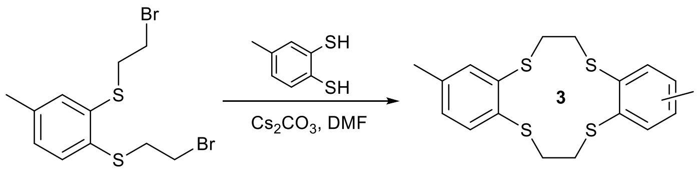

In a 250 ml round-bottom flask, 100 ml of DMF and 2.0 g (6.0 mmol) of Cs_2CO_3 were placed. The contents of the flask were kept at 55 °C. A 100 ml solution of 1.48 g (4.0 mmol) of 1,2-bis(2-bromoethylthio)-4-methylbenzne and 0.63 g (toluene-3,4-dithiol) were added within 3 days. After that, the mixture was stirred at 55 °C for another 3 days, and filtered. The solvent was removed on a rotary evaporator. Chromatographic separation (SiO_2, hexane:DCM gradient) yielded 0.76 g (Y=52 %) of viscous, colourless oil of compound **3**.

Compound 3: ^{1}H NMR (400 MHz, $CDCl_3$) δ 7.28 (dd, J = 7.8, 1.8 Hz, 2H), 7.19 (d, J = 2.3 Hz, 2H), 6.92 (ddd, J = 8.1, 4.2, 2.0 Hz, 2H), 3.34 – 3.27 (m, 8H), 2.26 (d, J = 2.7 Hz, 6H). ^{13}C NMR (101 MHz, $CDCl_3$) δ 138.02, 137.78, 137.76, 137.67, 134.10, 133.84, 133.63, 133.56, 133.26, 133.20, 128.43, 128.34, 34.04, 34.02, 33.72, 33.68, 20.96, 20.94.

2. NMR spectra

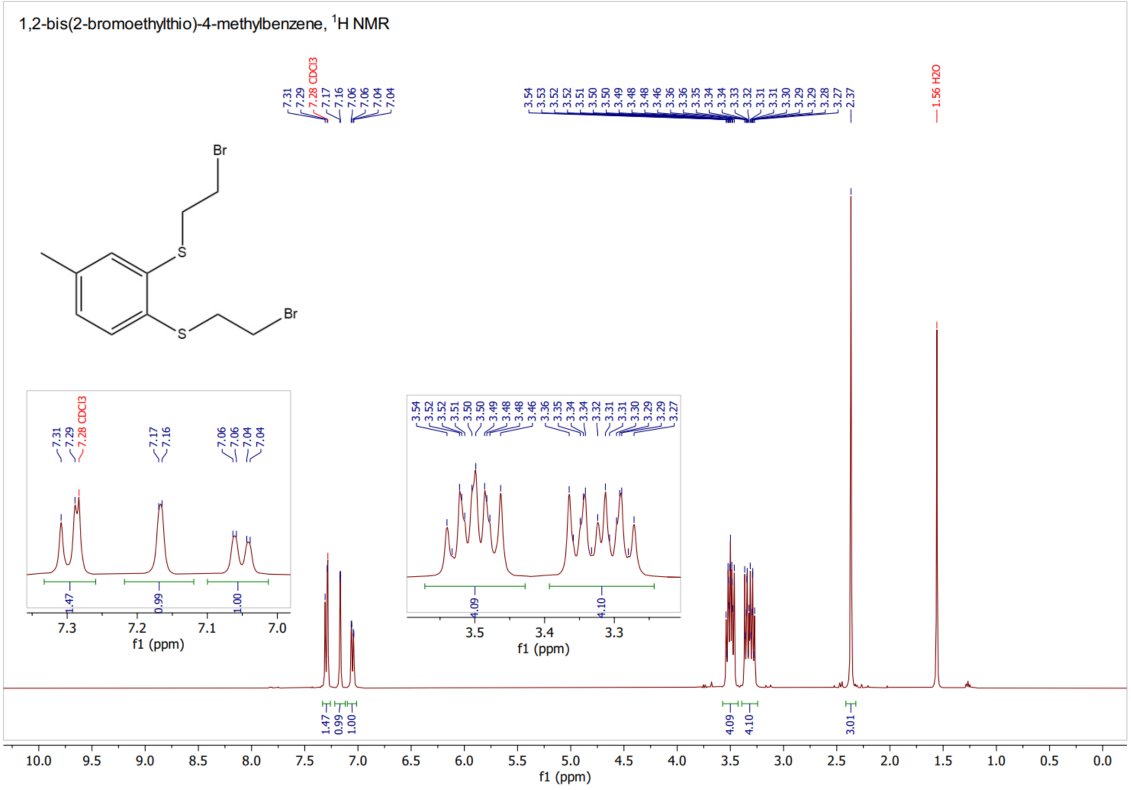

Figure S1. ^1H NMR spectrum of 1,2-bis(2-bromoethylthio)-4-methylbenzene.

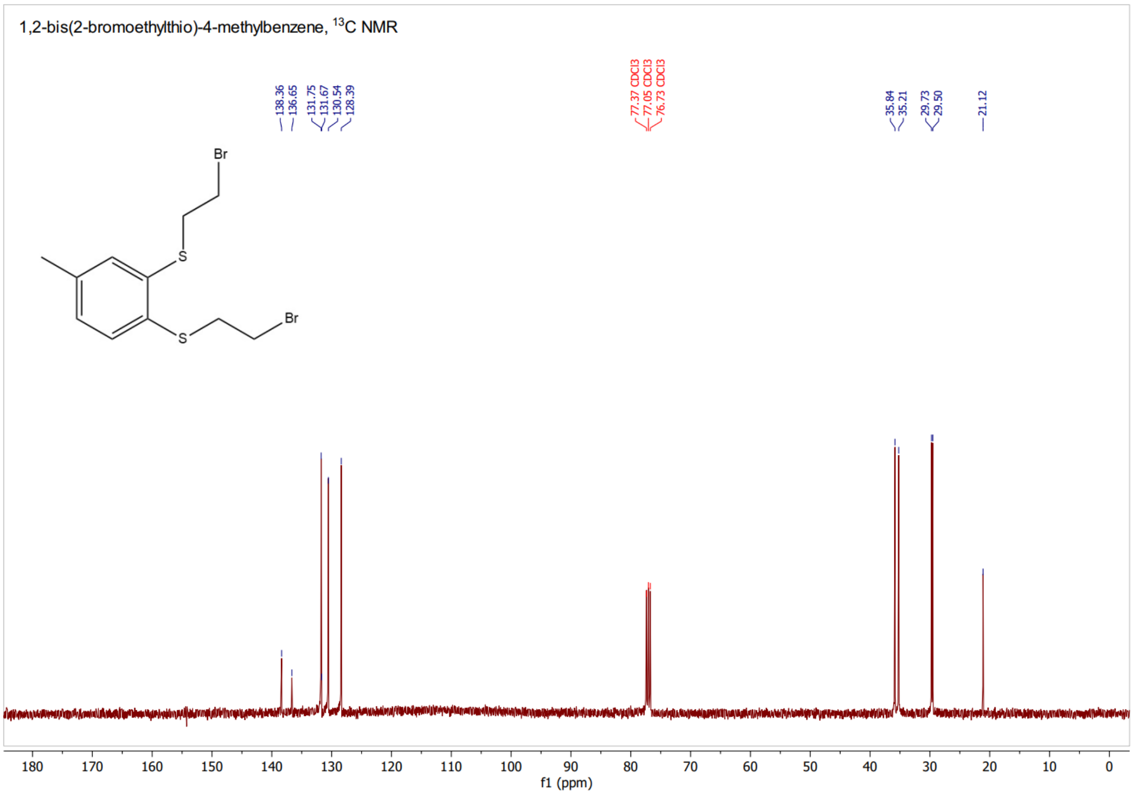

Figure S2. ^{13}C NMR spectrum of 1,2-bis(2-bromoethylthio)-4-methylbenzene.

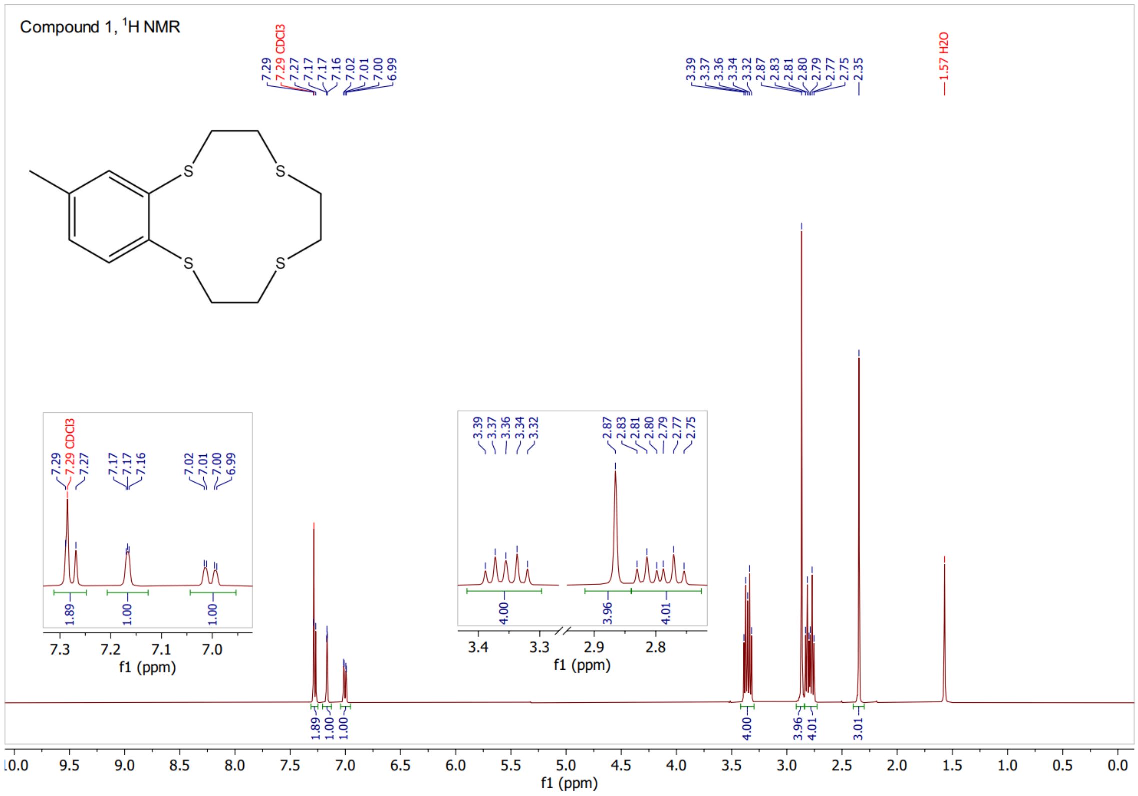

Figure S3. ^{1}H NMR spectrum of compound **1**.

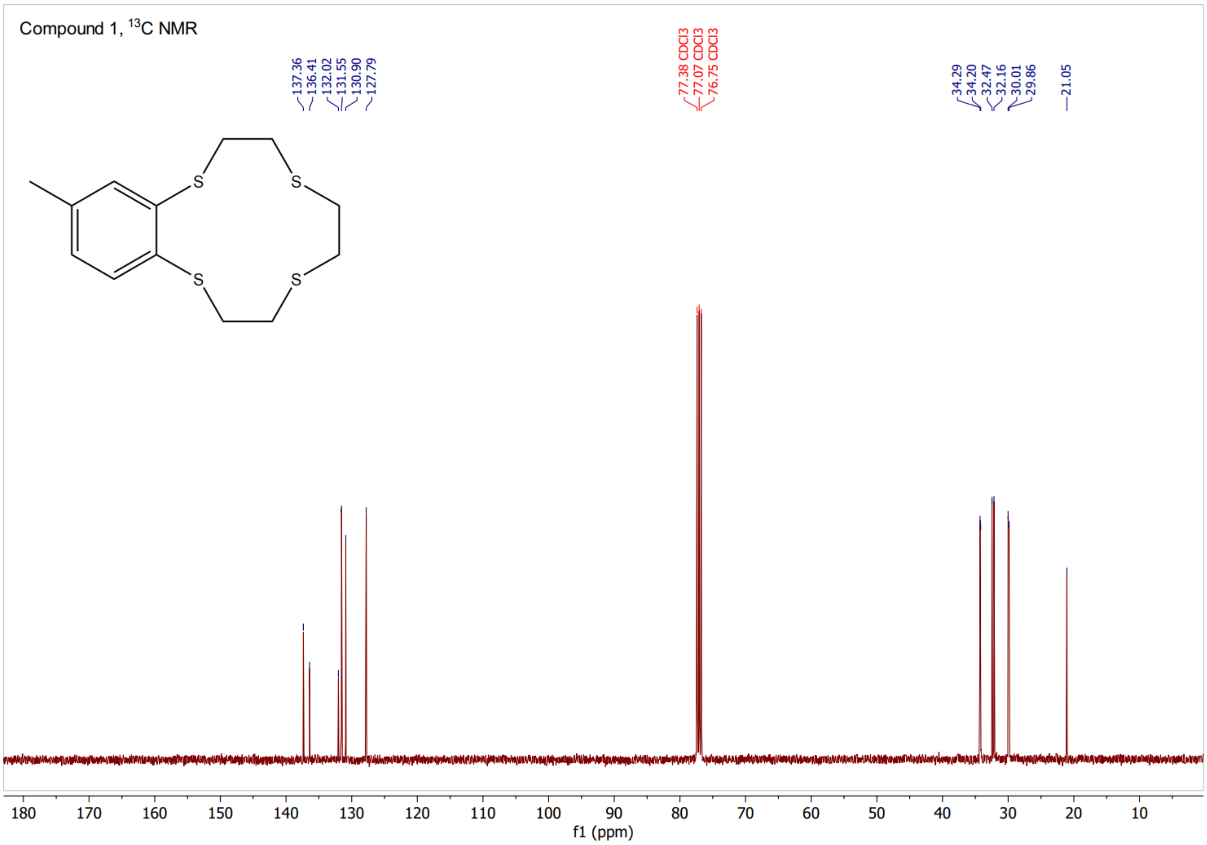

Figure S4. ^{13}C NMR spectrum of compound **1**.

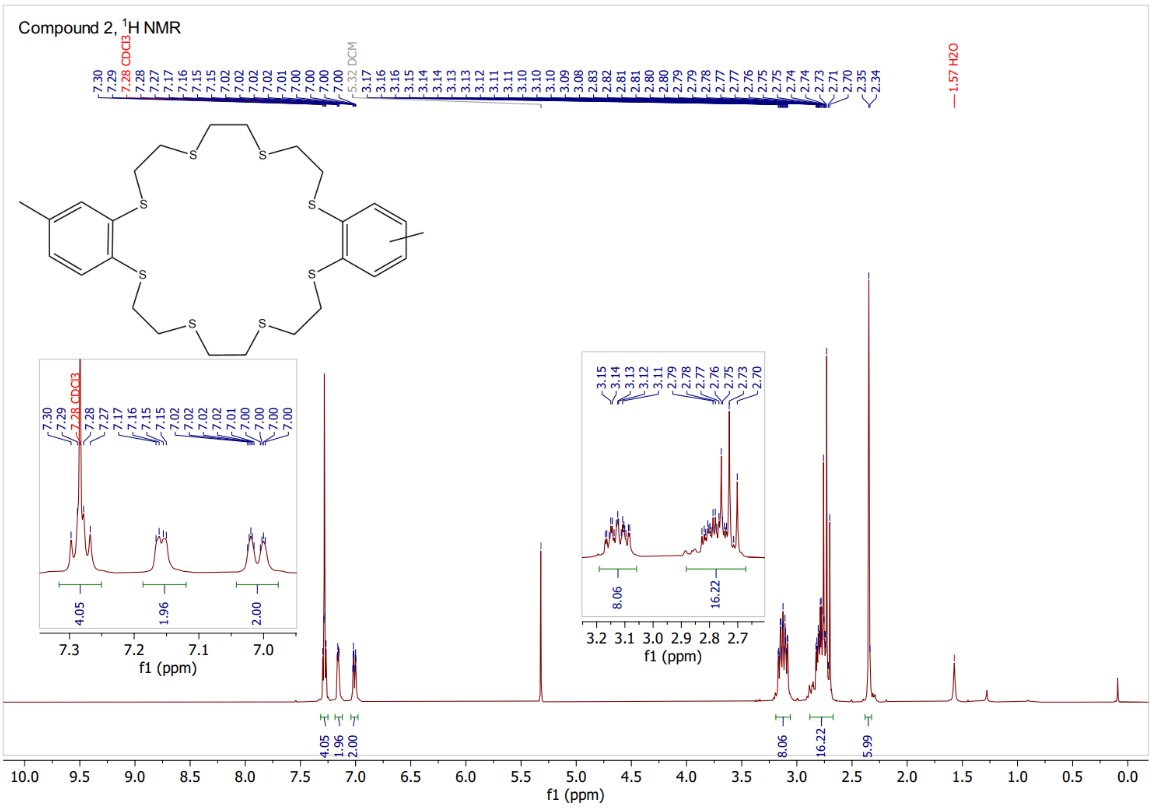

Figure S5. ^{1}H NMR spectrum of compound **2**.

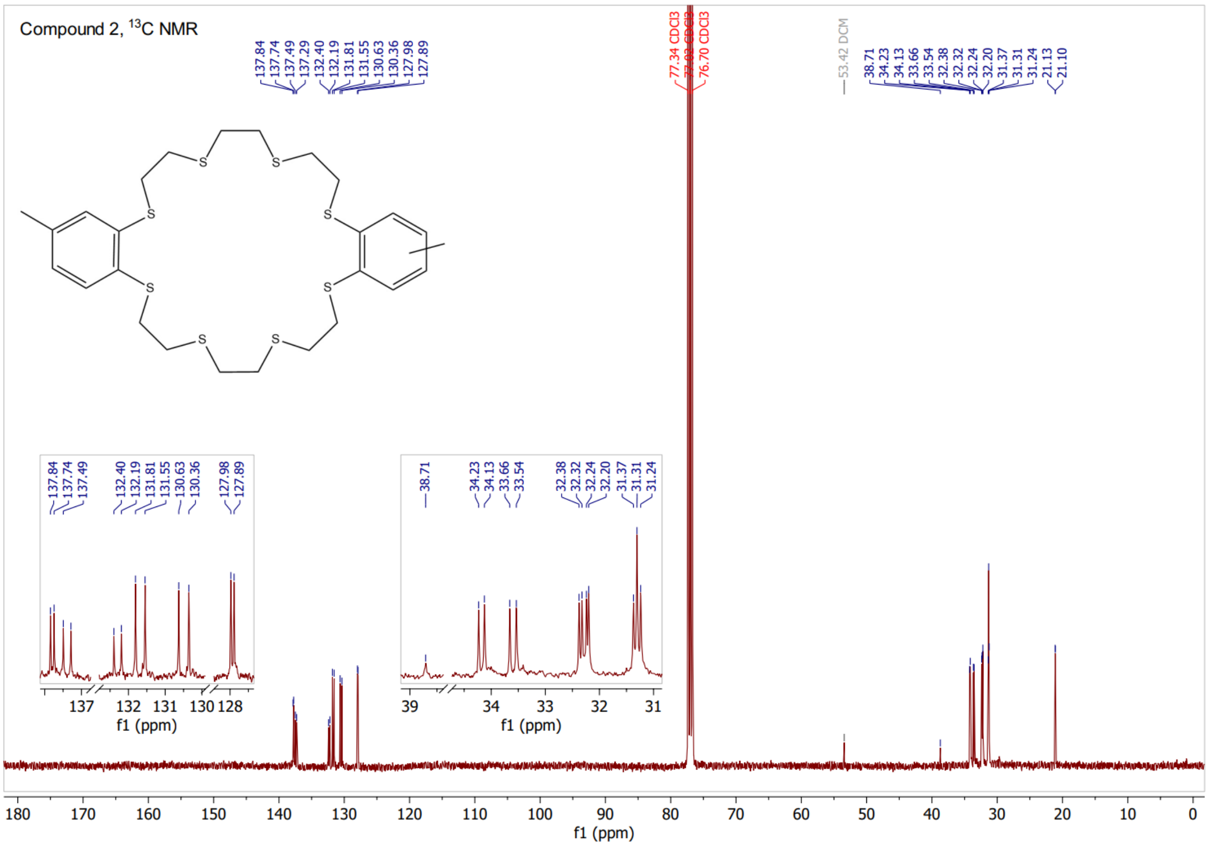

Figure S6. ^{13}C NMR spectrum of compound **2**.

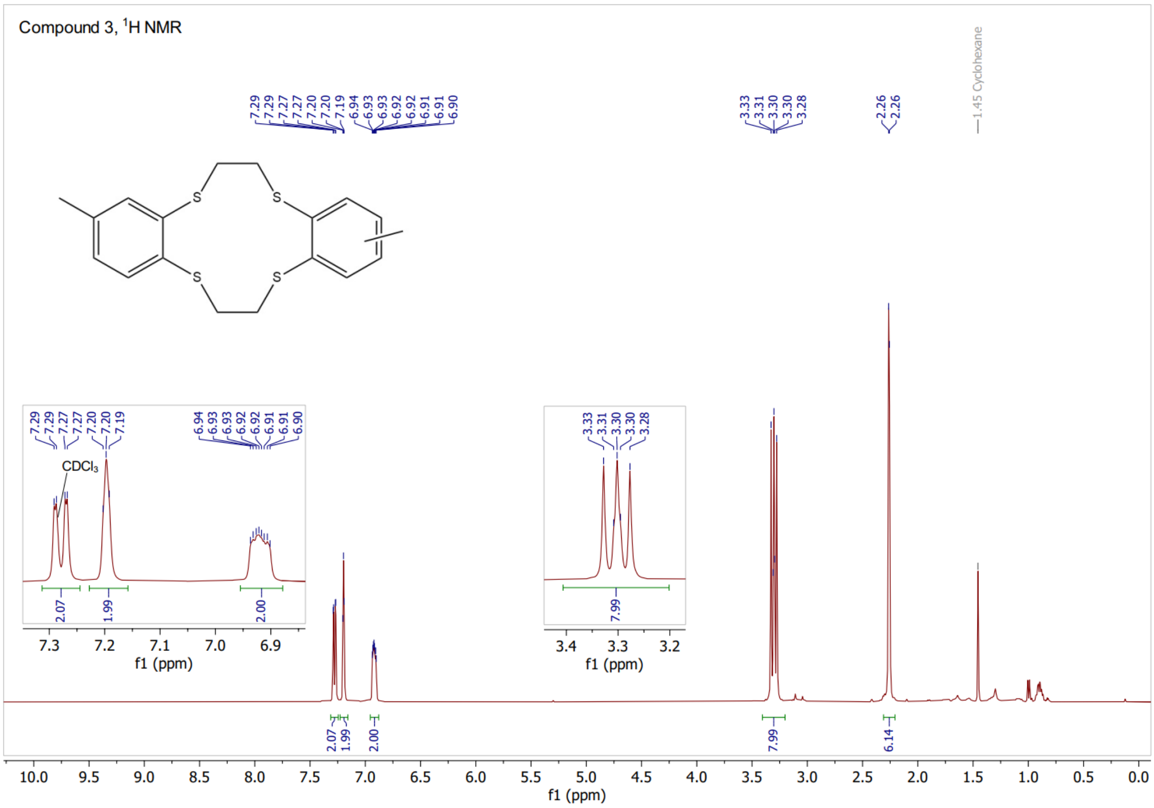

Figure S7. ^{1}H NMR spectrum of compound **3**.

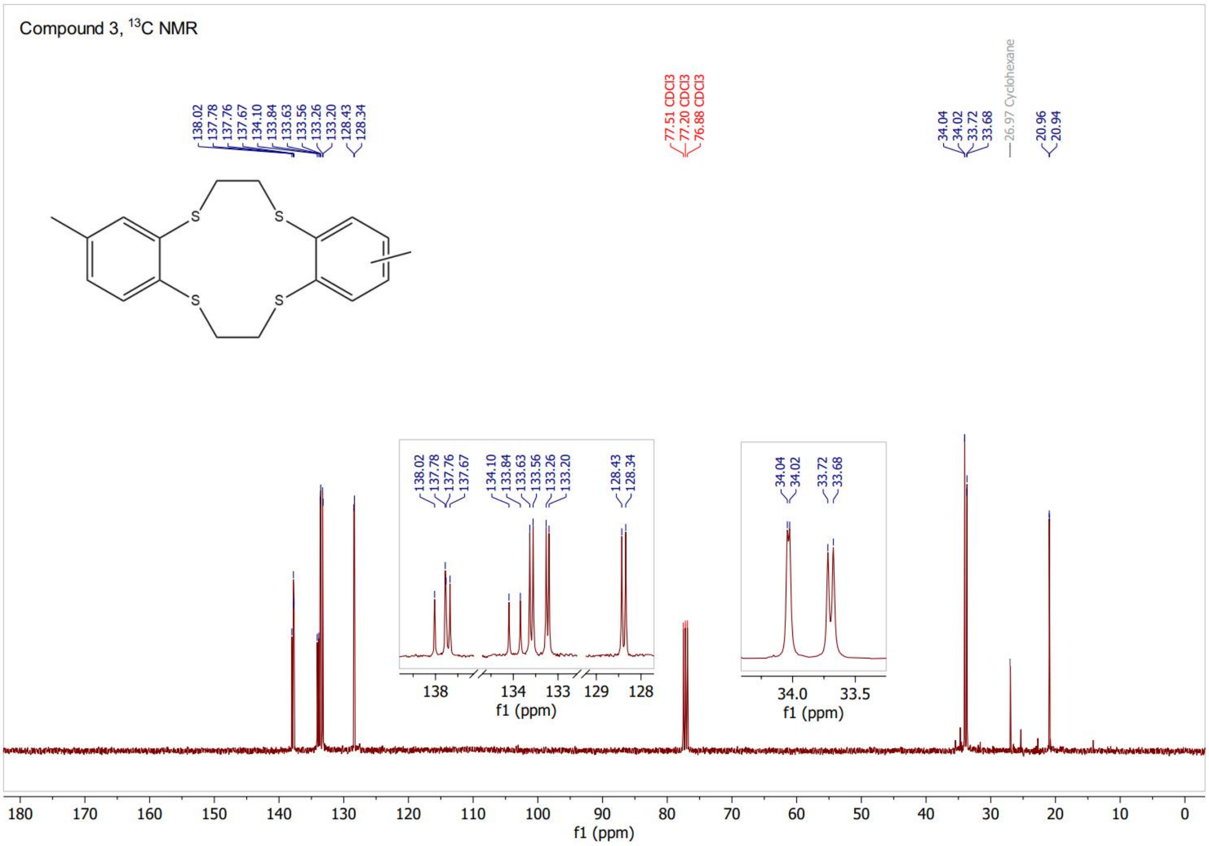

Figure S8. ^{13}C NMR spectrum of compound **3**.
